# Handedness in low-birthweight children: Insights in lateralization

**DOI:** 10.3389/fpsyg.2022.1018913

**Published:** 2023-01-11

**Authors:** Miriam Ittyerah

**Affiliations:** Department of Psychology, University of Delhi, New Delhi, India

**Keywords:** low-birthweight, lateralization, handedness, degree of handedness, children

## Abstract

Low-birthweight (LBW) children (*n* = 96) weighing less than 2.5 kg at birth and normal birthweight (NBW) children (*n* = 96) from Delhi, India, between the ages of 5 and 12 years were assessed for intelligence with Ravens Colored Progressive Matrices (RCPM), their handedness and hand proficiency for unimanual and bimanual performance. The objective was to know if there is a relation between birthweight and the development of handedness. Compared with NBW children, the LBW group had lower percentile scores for the RCPM. The LBW children were less lateralized than the NBW children in the hand preference test. The LBW children were faster than the NBW for sorting objects with each hand separately, but they were slower in the bimanual envelope task. This indicates a delay in interhemispheric transfer and the development of the corpus callosum that connects the cerebral hemispheres to enable bimanual coordination. In the absence of more direct evidence, hand skill was used as an index of the extent of lateralized control for performance. Findings indicate a relation between birthweight and lateralization in children tested for hand preference.

## Introduction

The WHO defines low-birthweight (LBW) as a birthweight of an infant of 2,499 g or less, regardless of gestational age. At the population level, the proportion of babies with LBW is an indicator of a multifaceted public health problem that includes long-term maternal malnutrition, ill health, hard work, and poor healthcare during pregnancy. Advances in neonatal care have resulted in improved survival of extremely low-birthweight (ELBW) infants (Norton, 2005). However, concerns have been expressed that these improvements may produce an increase in neurodevelopmental morbidity among survivors. Studies suggest children who are LBW are more likely to have lower IQ and academic achievement scores and require more educational assistance than children born at term because they experience greater difficulties at school (e.g., Bohm et al., 2002; Chaudhari et al., 2004), during adolescence, and adulthood (Madzwamuse et al., 2015). Oudgenoeg-Paz et al. (2017) observed a relation between early motor development and later cognitive outcomes in children born preterm or LBW. They found that the quality of general movement and postural control early in life is predictive of later cognitive outcomes.

In India, nearly 20% of neonates have LBW (Premananda et al., 2011), with higher frequencies of LBW among women than men. Birthweight is a critical factor in child development (Ortiz-Mantilla et al., 2008; Oudgenoeg-Paz et al., 2017). A question of interest is the relation between birthweight and the development of handedness. General movement is important for development (Einspieler et al., 2016), while children reach for objects in space and reveal an early emergence of hand preference (Corbetta et al., 2006). Although initially inconsistent (Scharoun and Bryden, 2014), a direction for the left or right hand emerges in 3 years, with adult-like performance emerging between 10 and 12 years. The degree of handedness that identifies how strongly one prefers one hand to the other stabilizes by approximately 8 years (McManus et al., 1988). Attempts to measure handedness have examined preference and performance (McManus and Bryden, 1992), indicating an increase in strength of hand preference with age. A right-hand preference has existed among 90% of humans over time (McManus, 2009), whereas meta-analysis reports on left-hand preference revealed a prevalence of 9.3–10.6% (Papadatou-Pastou et al., 2020) for different manual tasks. Early-life factors associated with hand preference confirm that infants with low-birthweight, being part of multiple births, and not being breastfed increased the probability of being left-handed (De Kovel et al., 2019). Heikkila et al. (2018) reported that low-birthweight is a risk factor for developmental delay and increases the chance of being left-handed.

Although a host of studies indicate the detrimental effects of children born preterm (Allotey et al., 2018) or low-birthweight, both at school performance and successive adolescence and adult periods (e.g., Chaudhari et al., 2004; Madzwamuse et al., 2015), none have tested their lateralization (cerebral hemisphere dominance) for hand preference and its relation to cognition and motor performance. Children with LBW often show motor impairments throughout childhood (De Kieviet et al., 2009; Johnson et al., 2015), and there is a large variability in the developmental trajectories of motor development in these children (Janssen et al., 2016). However, a relationship between hand preference/hand skill with cognition has not been tested in LBW children. It is of interest in the present study to know if the hand preferences of LBW and NBW children differ. Since children born preterm or LBW have been found to have motor impairments over periods of development (De Kieviet et al., 2009; Fuentefria et al., 2017) and lower academic achievement scores (Chaudhari et al., 2004; Hack, 2006), it is hypothesized that the LBW children in the present study may be less lateralized for hand preference and score lower on Ravens Colored Progressive Matrices (RCPM) and consequently suggest an effect of hand preference/Ravens score on reaction times in the unimanual/bimanual tasks.

## Methods

### Participants

Two groups of children, one a normal birthweight group (NBW) and another group of low-birthweight children (LBW), were selected for participation in the study. The children attended school regularly and were eager to participate after giving informed consent. There were 96 NBW children, 12 at each age level between 5 and 12 years, with six girls and six boys at each age. The inclusion criteria were full-term infants with a NBW of 2,500 g or more and a gestational age ranging from 37 to 41 weeks. The birthweight of the NBW group ranged between 2.5 and 3 kg, with a mean weight of 2.6 kg.

The LBW children were contacted with help from resources. Each child’s age, date of birth, and birthweight were verified after the parents gave consent for participation. The mean birthweight of the group was 2 kg (2,101.354 g for boys, SD 45.841 and 1,970.290 g for girls, SD 44.388), and ranged between 1,000 and 2,400 g for boys and 1,500 and 2,400 g for girls. All the LBW children were full-term infants. The inclusion criteria were full-term infants with a gestation age of 37 weeks and birthweight of fewer than 2,500 g. There were 96 LBW children, 12 at each age level, six girls and six boys, between the ages of 5 and 12 years. After participation in all the tasks, each child was rewarded with refreshments. The children belonged to lower-middle socioeconomic families.

The study was approved by the Ethical Committee at the Department of Psychology, University of Delhi. The study complies with all regulations and confirmation that informed consent was obtained. The study complies with the current Indian laws governing research with human subjects and the Helsinki Declaration of ethical principles involving research with human subjects (2013).

### Test materials

#### Ravens colored progressive matrices (1956)

Ravens CPM measures clear-thinking ability (Ravens, 1956). Before the ability to reason by analogy has developed, or in cases where intellectual ability has become impaired, the CPM can be used to assess the degree to which children and adults can think clearly, or the level to which their intellectual abilities have deteriorated (Ravens, 1956). Ravens CPM items are arranged to assess cognitive development up to the stage when a person is sufficiently able to reason by analogy and adopt this way of thinking as a consistent method of inference. This stage in intellectual maturation appears to be one of the earliest to decline as the result of organic dysfunction (Ravens, 1956). Three sets of 12 items are arranged to assess the chief cognitive processes of which children under 11 years of age are usually capable. Ravens CPM produces a single raw score that can be converted to a percentile based on normative data collected from various groups.

#### Handedness test

The handedness test comprised 16 items that are to be performed either unimanual or with assistance from the non-preferred hand. The items were taken from Ittyerah (1993, 2009) and consisted of suggestions from Geschwind in Healey et al. (1986). Geschwind and Galaburda (1985) suggested that the neural systems that control various aspects of manual preference may be independently lateralized. The handedness test required performance actions (Ittyerah, 1993, 2009). Performance tests provide more reliable responses in children than handedness questionnaires.

The home handedness questionnaire (HHQ) (Nelson et al., 2019) has a series of items that assess both unimanual and bimanual preferences in preschool children. The test identified a majority of children as right-handed. However, it was also sensitive to inconsistent/mixed preference in a fourth of the children, suggesting changes in hand-use patterns in children between 2 years and 10 months and 3 years and 8 months. In the present study, it was of interest to know the incidence of inconsistent hand preferences in both groups of children and if LBW is a cause for inconsistent hand preferences.

The 16 items in the handedness test are as follows:

1. Draw a line on paper (unimanual).
2. Touch your nose (unimanual).
3. Put pins on a plate (unimanual with minor assistance from the non-preferred hand).
4. Pick up buttons and place them on a plate (unimanual).
5. Cut paper with a pair of scissors (unimanual with major assistance from the non-preferred hand).
6. Open a jar (unimanual with assistance from the non-preferred hand).
7. Wind wool on a knitting needle (unimanual with major assistance from the non-preferred hand).
8. Throw a ball (unimanual with the involvement of axial musculature). The ball was approximately the size of a tennis ball and could be grasped by all the children.
9. Fly a paper arrow (unimanual).
10. Hit with a cricket bat (bimanual with the involvement of axial musculature). A small cricket bat was placed at the feet of the child, and the hand used to pick and hold the bat at the nearer end of the bat (away from the top of the handle) was considered to be the preferred hand.
11. Cut with an axe (the child must pretend that the cricket bat placed at the feet of the child is an axe and use it to cut wood).
12. Beat time to the music (emotional task). The child was presented with a rhyme set to music for a period of 30 s. The child was required to tap the table with her/his fingers in time with the music.
13. Snap your fingers (cognitive task involving distal musculature).
14. Dial a telephone (the child was presented with a toy telephone that could be dialed with the forefinger. The finger could be inserted into the space provided for the desired number and moved in a semicircle).
15. Pick up a pen (light object).
16. Pick up a travel bag (a relatively large and heavy object). This was a travel bag (10 × 18 inches) that contained some paper and toys. It was light enough to be picked and held by the youngest children.

#### Sorting task (unimanual)

This task consisted of sorting four different items that were mixed and presented on a tray. There were 15 bangles with a diameter of one inch, 15 colored beads, 15 butterfly buttons, and 15 blue buttons with a diameter of half an inch.

#### Envelope task (bimanual)

The task consisted of holding an envelope to pick and put items in it and pick and place the items in the envelope on a table before the child. It involved the use of both hands, one for holding the envelope and the other for putting/picking items it contained. Along with the envelopes, there were five small pictures of flowers or animals pasted on cardboard of half an inch square that were laid on a table to be picked or placed.

### Procedure

#### Ravens colored progressive matrices

The child was instructed to participate in the study by telling her/him that she would be playing a game. The first game was to perform the (RCPM) that was administered individually to each child. The child was instructed to point at the correct alternative for each of the items in the test. Each test was scored for the number of correct responses, and the child was assigned a percentile score according to norms (Deshpande and Ojha, 2002). All the percentile scores of the LBW and NBW children were recorded. The time taken to perform the CPM was recorded in seconds for each child.

#### Handedness test

The handedness test followed Ravens test. The child sat at a table before the experimenter and was instructed to perform each task. Each item in the test was placed before the child at her/his midline so that there was no left or right-side leaning bias for any item. In the first task, for example, draw a line on paper, a sheet of paper placed on the table was aligned to the midline of the child’s body, and a pencil was placed vertically on the paper at her/his midline. The paper was held in place by the experimenter. The child was required to take a pencil and draw a line. The hand used to draw the line was considered to be the preferred hand. In this manner, each task was performed, some of which were unimanual and others requiring major or minor assistance from the non-preferred hand. Each of the 16 actions was performed by every child at least twice at random. If a child consistently used a hand, for example, the right hand for drawing a line on the first and the second trials, it was scored as a right-hand preference. But if the child initially used the right hand and then the left hand on the second trial, a third trial was administered to test for consistency. Most children were consistent in their hand preferences, although there were some instances of Right Left Right (RLR) or Left Right Left (LRL). Though a third trial was not required, it was administered to know if the inconsistency was repeated consecutively. Even if two trials out of three are performed with the same hand, the hand actions are inconsistent. Of the 16 actions in the handedness task, four tasks revealed inconsistent actions in both groups. They were to draw a line (2 LBW and 3 NBW), touch your nose (16 LBW and 21 NBW), open a jar (4 LBW and 3 NBW), and wind wool (4 LBW and 2 NBW). Inconsistent hand actions were scored as non-preferred hand actions.

The hand preferences were converted to a laterality index (LI) (McManus et al., 1988) derived from the performance scores of each child. Scores for direction and degree were calculated for each subject. The scores range from −1 through zero to + 1, and those scoring greater than zero were taken as right-handers. The degree of lateralization was considered to be the absolute value of LI (range 0–1). The formula for calculating the LI is as follows: (R−L)/(R + L) × 100. The distributions of hand preference are presented in Figures 2, 3.

**FIGURE 1:**
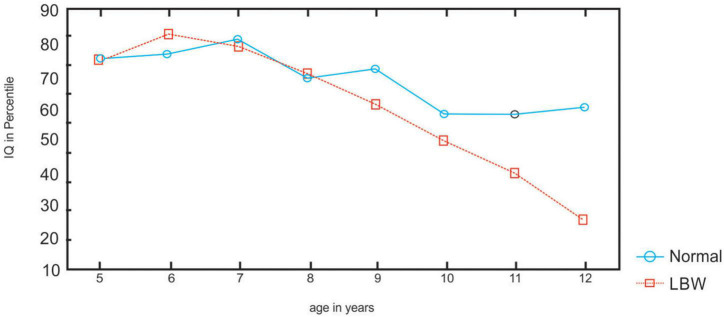
Percentile scores of the LBW and NBW children on Ravens test.

**FIGURE 2:**
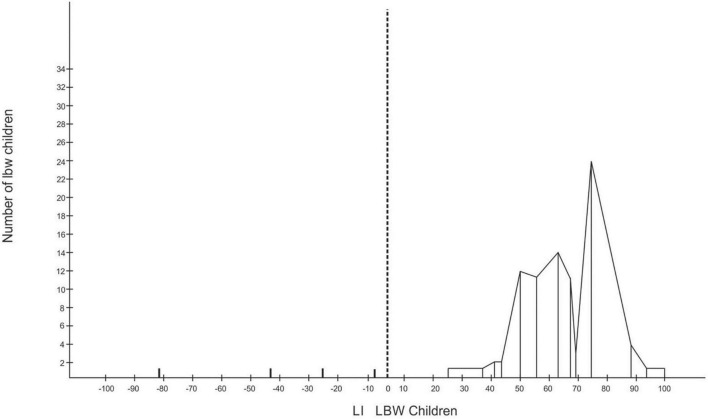
Laterality index (LI) for low-birthweight (LBW) children.

**FIGURE 3:**
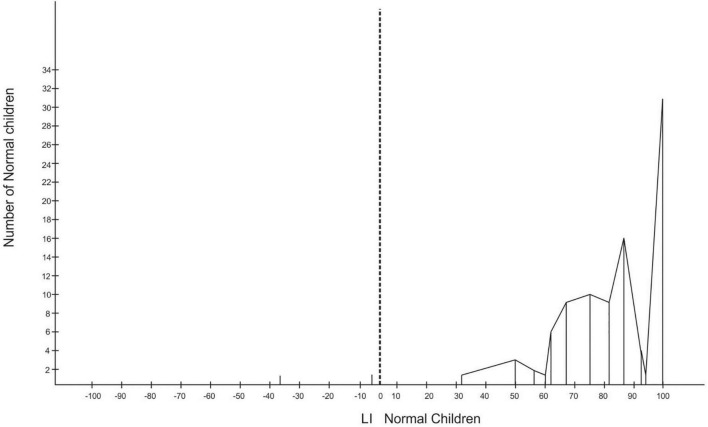
Laterality index (LI) for normal birthweight (NBW) children.

#### Sorting task (unimanual)

The child sat before a table to sort mixed items presented on a tray. S/he was instructed to pick each item in the tray with one hand only and place it in one of the four containers meant for each group of items. The time taken to sort the items into groups was recorded in seconds for each hand separately. The hand used for sorting was counterbalanced across children in both groups by ensuring that if a child began sorting with her/his right hand initially and the left hand followed, the next child was instructed to begin sorting with her/his left hand followed by the right hand.

#### Envelope task (bimanual)

The child was presented with two envelopes at a table, one yellow and another purple, and instructed to put the pictures laid on the table into an envelope (yellow) and place the envelope on the table. The child was then required to pick each picture in the envelope and replace it on the table along with the envelope. This procedure was repeated with the purple envelope. The time taken to pick and place the pictures in the envelope and replace them on the table was recorded in seconds for each of the colored envelopes. This procedure was repeated so that each child performed the task twice with different envelopes each time.

## Results

Of the 96 LBW children, four were left-handers with absolute LI scores, while the rest were right-handers. There were two left-handers with absolute LI scores among the NBW children (refer to Figures 2, 3).

To know the incidence of mixed-handers, the LI were grouped into three, with LI ranging between −100 and −60 for left-handers, −60 and +60 for mixed-handers, and +60 and 100 for right-handers. The grouping revealed that there was only one left-hander with an LI of −81 among the LBW, 33 mixed-handers among the LBW, and eight mixed-handers in the NBW group. The rest were right-handers with an LI of 60 or more. The chi-square test revealed that the frequency of non-right-handers among the LBW differs significantly from the frequency of non-right-handers in the NBW group [*X*^2^ = 19.75, df (1), *p* < 0.01]. To determine the handedness strength, laterality quotients (LQ) for each group revealed that the LBW have an LQ of 94.48, the NBW an LQ of 98.75 and the LQ for both groups together is 96.89.

Group means and SD for Ravens test (M. LBW: 60.1 SD: 22.62; M. NBW 68.49, SD: 18.19), LI (M. LBW: 59.95, SD: 20.38; M. NBW: 81.44; SD: 17.66), left-hand sort (M. LBW: 67.24 s, SD: 11.28 s; M. NBW: 72.58 s; SD: 14 s), right-hand sort (M. LBW: 61.44, SD: 10.77; M. NBW: 72.08 s, SD: 15.43), and the bimanual task (M. LBW: 62.01 s, SD: 13.23; M. NBW: 47.21 s, SD: 10.74 s) indicate that the performance of the LBW children is weaker than that of the NBW in all tasks, except for unimanual sorting where the LBW is faster with each hand.

A multifactorial analysis of variance using Statistica computed for gender (2) group (2) and age (8) with repeated measures on the tasks indicate that the main effect of gender is not significant [*F*_(5,155)_ = 1.59; *p* < 17] indicating that girls and boys do not differ on the tasks. The main effect of group is significant [*F*_(5,155)_ = 29.15; *p* < 0.0000], indicating that the LBW and NBW children differ on all the tasks.

The main effect of age is significant [*F*_(35,654)_ = 8.2; *p* < 0.0002]. Children differ with age in performance. The group × age interaction is significant [*F*_(35,654)_ = 2.15; *p* < 0.0002]. *Post-hoc* tests (Newman–Keuls) revealed that the LBW children scored lower than the NBW in Ravens test at ages 11 and 12 years (*p* < 0.05) (refer to Figure 1).

**FIGURE 4:**
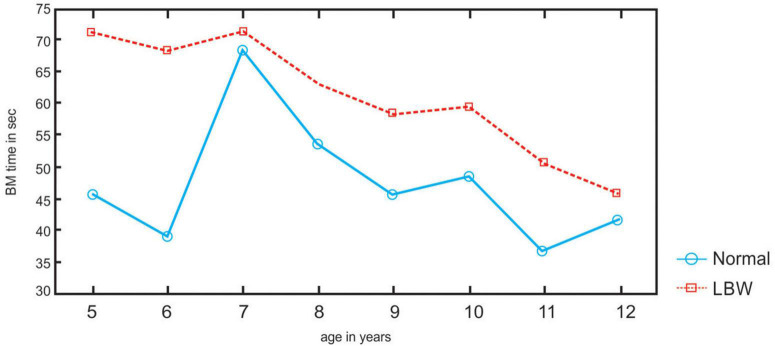
Bimanual coordination times in LBW and normal birthweight (NBW) children.

The LBW at ages 8–12 years are less lateralized in their hand preference than the NBW (*p* < 0.05) (refer to Figures 2, 3). LBW are faster than the NBW for sorting with their left and right hands (*p* < 0.05), whereas LBW are slower than the NBW in the bimanual task at all ages (*p* < 0.05) (refer to Figure 4). In the bimanual task, the envelope was held in the right hand by ten LBW children, while the picking and sorting were done with their left hands. Among the NBW children, the envelope was held in the right hand by eight children, while the picking and sorting were done with their left hands. The remaining children held the envelope in their left hands and sorted with their right hands. The chi-square test revealed no differences between the groups in the division of labor between the hands [*X*^2^ = 0.25, df (1), p, n.s.].

In order to test whether LI related to the subject’s ability on the task, correlations were computed between LI and the task. Correlations were significant only for LI and Ravens IQ, *r* = −0.50, *p* < 0.05, and LI and bimanual coordination, *r* = −0.23, *p* < 0.05, indicating a negative correlation between LI and task. Ravens scores were higher, and bimanual coordination times were faster for the NBW than the LBW children.

## Discussion

The results indicate that the performance of LBW children differs from that of NBW children in all the tasks. The LBW children scored lower than the NBW children for reasoning in Ravens test, which is a measure of general intelligence. Furthermore, the percentiles of LBW children decline at 11 and 12 years, indicating a decline with age in intellectual performance. The decline in performance has been associated with nutritional status among school children aged 9–10 (Zaini et al., 2005), with non-verbal intelligence among children with lead exposure (Counter et al., 2005), and for reasoning in Ravens colored matrices in hearing-impaired children (Weichbold and Herka, 2003). Others, for example, Hack (2006), reported that educational disadvantage associated with very LBW persists into early adulthood. Assessments conducted at 20 years of age for education, cognitive and academic achievement, and risk-taking showed that very few LBW adults when compared with NBW adults, had graduated from high school. Furthermore, children born preterm/LBW perform below term-born infants in executive functions (van Houdt et al., 2019) and social competence (Ritchie et al., 2015; Taylor, 2020). The results of the RCPM indicate a detrimental prognosis for LBW children with a decline in scores, particularly at ages 11 and 12 years.

The LI indicates that the LBW children are less lateralized for handedness than the NBW children. There are more numbers of non-right-handers (34%) among the LBW children than in the NBW group (8%). The findings reveal a relationship between birthweight, hand preference, and general intelligence tested by the RCPM. Marlow et al. (2019) found preterm children (25 weeks of gestation) were less lateralized for hand preference, being 28% non-dominant for right-hand use compared with NBW children, that were 10% non-dominant for right-hand use. Meta-analysis revealed that children born preterm had a twofold increase in odds for non-righthandedness when compared with full-term control children (Domellöf et al., 2011). The meta-analysis showed an association between preterm birth and non-righthandedness in children aged 3–18 years and suggested that preterm birth is associated with an early disturbance in the development of an asymmetrical brain which supports stable handedness and lateralization. Evidence indicates that when handedness is unstable or mixed, lateralization is reduced and associated with reduced cognitive function (Yeo et al., 1997, 2007; Crow et al., 1998; Leask and Crow, 2005). Very low-birthweight (VLBW) children are at risk of structural brain abnormalities and neurocognitive deficits (Farajdokht et al., 2017) and prone to perinatal brain injury with an increased risk of developing motor and cognitive impairments during childhood and adolescence (Volpe, 2009; Williamson et al., 2015). Schmitz et al. (2017) reported the need to integrate genes and the environment to comprehend the ontogenesis of handedness. For example, the hand actions of children may be shaped by parental influence and, thus, mediated by epigenetic mechanisms (Laland, 2017).

The LBW children are faster than the NBW for unimanual sorting with their left and right hands. Although the LBW are less lateralized than the NBW in the hand preference task, they are quicker than the NBW with both hands for sorting objects. Although there is an inherent specialization such as better movement processing with the right hand (De Renzi et al., 1983) or better spatial detection with the left hand (De Renzi, 1978), equal proficiency of the left and right hands has been demonstrated (Millar, 1997; Ittyerah, 2013) with complementarity between the hands during task performance (Millar and Al-Attar, 2003a,b).

The LBW children are slower than the NBW children in the bimanual envelope task. Equal hand ability differs from bimanual coordination. In bimanual tasks, both hands must work together even when the movements of each hand or their role in the overall movement differs. Thus, they are typically more demanding than unimanual tasks (Serrien et al., 2014). Although there is an early instability in hand preference (Carlson and Harris, 1985) for reaching during infancy, reaching is usually performed bimanually (Morange-Majoux and Devouche, 2019), indicating the role of interhemispheric pathways. The development of coordinated bimanual behavior in children continues for years, reflecting experience along with the maturation of critical brain areas, such as the corpus callosum, which supports the interhemispheric transfer of information necessary for bilateral coordination (Fagard et al., 2001; De Boer et al., 2012; Gooijers and Swinnen, 2014). Furthermore, the performance of young children in a peg-moving task did not slow (Bradshaw et al., 1988) when crossing the body midline, indicating this observation is incompatible with the notion of callosal immaturity in young children, whereas bimanual performance lagged in patients with brain injury (Pixa and Pollok, 2018).

A limitation of the study is that foot preferences were not assessed. Evidence shows that foot preferences in children take longer to stabilize than hand preferences (Musalek, 2015). Testing preferences between the groups for footedness, as in handedness, may provide additional information related to development.

In summary, the findings of the study indicate a relation between birthweight and the development of handedness and its relation to cognition and motor performance. Low-birthweight deteriorated performance in the Ravens test, revealed weaker lateralization of hand preferences, and slowed bimanual coordination in the envelope task.

## Data availability statement

The raw data supporting the conclusions of this article will be made available by the author, without undue reservation.

## Ethics statement

The studies involving human participants were reviewed and approved by the Department of Psychology, University of Delhi, India. Written informed consent to participate in this study was provided by the participants or their legal guardian/next of kin.

## Author contributions

The author confirms being the sole contributor of this work and has approved it for publication.
